# Alterations of the Lipid Metabolome in Dairy Cows Experiencing Excessive Lipolysis Early Postpartum

**DOI:** 10.1371/journal.pone.0158633

**Published:** 2016-07-06

**Authors:** Elke Humer, Annabella Khol-Parisini, Barbara U. Metzler-Zebeli, Leonhard Gruber, Qendrim Zebeli

**Affiliations:** 1 Institute of Animal Nutrition and Functional Plant Compounds, Department for Farm Animals and Veterinary Public Health, University of Veterinary Medicine Vienna, Vienna, Austria; 2 University Clinic for Swine, Department for Farm Animals and Veterinary Public Health, University of Veterinary Medicine Vienna, Vienna, Austria; 3 Institute of Livestock Research, Agricultural Research and Education Centre Raumberg-Gumpenstein, Irdning, Austria; University of Maryland, UNITED STATES

## Abstract

A decrease in insulin sensitivity enhances adipose tissue lipolysis helping early lactation cows counteracting their energy deficit. However, excessive lipolysis poses serious health risks for cows, and its underlying mechanisms are not clearly understood. The present study used targeted ESI-LC-MS/MS-based metabolomics and indirect insulin sensitivity measurements to evaluate metabolic alterations in the serum of dairy cows of various parities experiencing variable lipolysis early postpartum. Thirty (12 primiparous and 18 multiparous) cows of Holstein Friesian and Simmental breeds, fed the same diet and kept under the same management conditions, were sampled at d 21 postpartum and classified as low (n = 10), medium (n = 8), and high (n = 12) lipolysis groups, based on serum concentration of nonesterified fatty acids. Overall, excessive lipolysis in the high group came along with impaired estimated insulin sensitivity and characteristic shifts in acylcarnitine, sphingomyelin, phosphatidylcholine and lysophospholipid metabolome profiles compared to the low group. From the detected phosphatidylcholines mainly those with diacyl-residues showed differences among lipolysis groups. Furthermore, more than half of the detected sphingomyelins were increased in cows experiencing high lipomobilization. Additionally, strong differences in serum acylcarnitines were noticed among lipolysis groups. The study suggests an altered serum phospholipidome in dairy cows associated with an increase in certain long-chain sphingomyelins and the progression of disturbed insulin function. In conclusion, the present study revealed 37 key metabolites as part of alterations in the synthesis or breakdown of sphingolipids and phospholipids associated with lowered estimated insulin sensitivity and excessive lipolysis in early-lactating cows.

## Introduction

Early-lactating dairy cows are not able to meet their energy needs through alimentary sources. Therefore, early lactating cows mobilize triacylglycerols (TAG) from adipose tissues to generate the necessary fuels; thus using nonesterified fatty acids (NEFA) as an energy source [1]. Lipolysis of adipose tissues is part of the metabolic adaptation of cows to counteract the acute energy deficit, which is orchestrated by endocrine changes including a lowered insulin sensitivity in peripheral tissues, i.e. the adipose tissues and skeletal muscles [2]. Although the exact underlying mechanisms are not fully understood yet, it is believed that the modified peripheral insulin sensitivity accelerates adipose tissue mobilization [3], leading to excessive lipolysis in many early-lactation cows [4–7]. The excessive rates of lipolysis result in markedly elevated plasma concentrations of NEFA, making preprandial NEFA an accurate indicator of the actual body lipid loss [8]. Moreover, postpartum NEFA has been shown to be the best predictor, i.e. compared to beta-hydroxybutyrate, of many metabolic diseases associated with excessive lipolysis in cows, including displaced abomasum, ketosis, and metritis [9].

A smooth metabolic adaptation during the transition period is of utmost importance to prevent excessive mobilization of fat tissues and consequently to improve animal health and productivity. We recently observed different metabolic adaptation strategies in periparturient dairy cows to cope with the energy deficit postpartum, despite similar feeding and management conditions [10]. Although these cows had similar milk production, body weight and body condition scores, they showed different levels of circulating NEFA and differed in the estimated sensitivity to insulin. Thus, the observed variability in lipolysis among individual cows seems to be related to other factors than nutrition and management.

Compared to traditional techniques that focus on single metabolites to describe lipolysis in cows, such as NEFA or beta-hydroxybutyrate, metabolomic technologies have allowed detection of classes of small-sized metabolites reflecting derailment of key metabolic pathways, which have advanced our understanding by better explaining the mechanisms behind metabolic disorders in cattle such as ketosis [11–13]. Given this potential, we used targeted ESI-LC-MS/MS-based metabolomics approach to get a more comprehensive view on the plethora of metabolites that change during the early lactation period in healthy cows experiencing excessive degrees of lipomobilization despite similar feeding and management conditions with low lipomoblization cows. In terms of selecting metabolites of interest for the study, phosphatidylcholines were primarily chosen, due to their pivotal role in lipid metabolism [14]. More specifically, they are essential for VLDL synthesis in the liver, thus being of crucial importance for the export of TAG [14, 15]. Furthermore, shifts in the phospholipidome have been previously linked to metabolic disorders related to insulin resistance in humans, whereby it has been suggested that especially PC with shorter chain lengths and double bonds trigger the development of disturbed insulin functions, although the detailed mechanisms remain unclear so far [16]. Thus, we hypothesized that changes in the serum phospholipidome would mirror different degrees of lipomobilization in early lactating cows experiencing impaired insulin sensitivity. In the present study a special emphasis was also put on sphingolipids, as they have been shown to be associated with disturbed insulin functions in obese humans [17], and recently also in dairy cows [6]. Based on these findings, we further hypothesized that alterations in the sphingomyelins likely coincide with the progression of insulin resistance in postpartal dairy cows. Another key aspect was the determination of several acylcarnitines, which was based on previously recognized associations with inflammaton, hyperlipidemia and insulin resistance in humans [18].

Thus, the objective of this study was to identify serum biomarkers, primarily belonging to the serum lipidome, that distinguish cows with high lipid mobilization from those with medium and low lipid mobilization, respectively, and to gain a deeper understanding of the alterations of the blood metabolome in relation to lipomobilization early postpartum before symptoms develop. The degree of lipolysis might also be influenced by parity, with first production heifers being differently sensitive to metabolic changes than multiparous cows that have a higher production potential. Therefore, an additional aim of this study was to evaluate differences in the serum metabolome between early-lactating primiparous and multiparous dairy cows.

## Material and Methods

### Animals, Housing, and Feeding

The trial was conducted at the Dairy Research Facilities of the Institute of Livestock Research, Agricultural Research and Education Centre Raumberg-Gumpenstein, Austria). The experiment was part of a larger study reported elsewhere [10]. Data of metabolic adapation of cows, energy and nutrient balance, and rumen pH during transition period are presented in that article. Details regarding diets, feed analyses, and feeding management of cows are given in that article, too. In brief, a total of 30 (12 primiparous and 18 multiparous) early lactation cows (25 Holstein and 5 Simmental) were used in this study. The mean lactation number was 2.56 for multiparous cows (10 cows in 2^nd^ lactation, 6 cows in 3^rd^ lactation, and 2 cows in the 4^th^ lactation). Primiparous cows weighed 580 ± 17.1 kg (mean ± SD), whereas the multiparous cows weighed 643 ± 17.1 kg. Holstein cows weighed 600 ± 11.5 kg and Simmental cows weighed 709 ± 36.6 kg. Average lactation number was 2.00 ± 1.22 in Holstein cows and 1.96 ± 0.93 in Simmental cows, respectively.

All cows were fed the same early-lactation cow diet and were under the same feeding management throughout the transition period [10]. Diet was based on forages of high quality such as second cut meadow hay, corn silage, and grass silage as well as ground barley and a protein-mineral-vitamin-supplement as concentrates (S1 Table). The diet was formulated according to nutritional recommendations of the German Society of Nutrition Physiology [19] for fresh-lactating dairy cows.

Cows were located in a free stall with straw bedding material and milked twice daily [10]. All procedures involving animal handling were in accordance with national regulations for animal use in research and approved by the Veterinary Directorate of Styria (Graz, Austria) according to §9ff, Law for Animal Experiments (GZ FA10A-78Gu-19/2012-2).

### Blood Sampling

On d 21 after parturition, blood samples were collected shortly before the morning feeding from the coccygeal vein using serum 10 mL vacutainer tubes (Vacuette, Greiner Bio-One, Kremsmünster, Austria). This samplig day was chosen based on a previous screeing among several measurement days during the transition period, showing the highest NEFA values as well as the largest differences among lipolysis groups at d 21 after parturition [10]. Samples were allowed to clot for 30 min at 25°C and serum was separated by centrifuging at 3,000 × g at 4°C for 20 min and subsequently stored at −20°C until analyses.

### Classification of Cow Groups according to Lipolysis

Cows were classified into 3 different lipolysis groups based on serum NEFA concentrations, using common risk thresholds for postpartal NEFA concentrations in dairy cows [9, 20, 21]. Cows were classified as low, medium, and high lipolysis group if serum NEFA concentrations were <0.4 mmol/L, between 0.4 to 0.7 mmol/L, and >0.7 mmol/L, respectively. These thresholds were chosen on the basis of previous studies, reporting that postpartal NEFA levels greater than 0.4 mmol/L are indicative of problems with moderate negative energy balance and subsequent lipolysis [21], whereas levels greater than 0.7 have been reported to indicate a severe negative energy balance, strong lipolysis [20], and to be likely associated with development of ketosis [22]. The low group comprised 10 cows (8 Holstein, 2 Simmental), the medium group 8 cows (8 Holstein), and the high group 12 cows (9 Holstein, 3 Simmental). The low group included 4 primiparous and 6 multiparous cows (mean lactation number 1.90 ± 0.94), the medium group 1 primiparous and 7 multiparous (mean lactation number 2.13 ± 0.60), and the high group 7 primiparous, 5 multiparous cows (mean lactation number 1.83 ± 1.07). Cows of the low group weighed 615.2 ± 67.6 kg, whereas the medium group cows weighed 609.1 ± 51.3 kg, and the cows of high group weighed 625.6 ± 85.5 kg.

### Measurement of Serum NEFA and estimated Insulin Sensitivity Response

Concentration of NEFA and glucose was measured by standard enzymatic colorimetric analysis using an autoanalyzer for clinical chemistry (Cobas 6000/c501; Roche Diagnostics GmbH, Vienna, Austria). In considering that lipolysis is modulated by insulin sensitivity [6], insulin was also determined using a bovine specific commercial ELISA kit according to the manufacturer’s instructions (Monobind Inc., CA, USA). The intra-assay and inter-assay coefficients of variance (CV) were controlled by limiting the coefficient of variation to ≤10% for insulin and < 5% for other blood variables. Insulin, glucose and NEFA concentrations were subsequently used to calculate the revised quantitative insulin sensitivity check index (RQUICKI) as RQUICKI = 1/[log (glucose) + log (insulin) + log (NEFA)], as described by Holtenius and Holtenius [23]. The RQUICKI was chosen as an indirect indicator of insulin sensitivity, and is referred as such in this paper to estimate the sensitivity to insulin response of the cows with differing lipolysis degrees.

Data of the blood variables during the transition period of the cows in the current study are reported in our companion paper [10]. Here, we use data of NEFA and estimated insulin sensitivity (i.e., RQUICKI) measurements to distinguish among groups and interpret these values in the context of altered metabolomic profile.

### Metabolome Profiling

In order to cover a broader spectrum of small-sized metabolites that belong or are interconnected to the sentinel blood parameters, metabolome profiling was performed using a targeted metabolomics approach based on electrospray ionization liquid chromatography–mass spectrometry (ESI-LC-MS/MS). The Absolute-IDQ platform (Kit p180, Biocrates Life Sciences AG, Innsbruck, Austria) was employed for targeted metabolite profiling as described by the manufacturer. This platforms allows a simultaneous detection of 217 metabolites, including 21 amino acids (proteinogenic amino acids, citrulline and ornithine), free carnitine (C0) and 40 acylcarnitines (Cx:y), hydroxylacylcarnitines [C(OH)x:y] and dicarboxylacylcarnitines (Cx:y-DC), the sum of hexoses, 15 sphingomyelins (SMx:y) and sphingomyelin derivatives [SM(OH)x:y], as well as 14 lyso-phosphatidylcholines and 76 phosphatidylcholines (PC). The latter were further differentiated with respect to the presence of ester (“a”) and ether (“e”) bonds in the glycerol moiety, whereby two letters “aa” (= diacyl) and “ae” (= acyl-alkyl) indicate that two glycerol positions are bound to a fatty acid residue, while a single letter “a” (= acyl) indicates the presence of a single fatty acid residue. The lipid side chain composition is abbreviated with “Cx:y”, whereby “x” denotes the number of carbons in the side chain and “y” the number of double bonds. A detailed list of all analyzed metabolites is presented elsewhere [24]. All analyses were performed by the TargetIDQ Service of BIOCRATES Life Science AG. The assay was based on phenylisothiocyanate(PITC)-derivatization in the presence of internal standards followed by FIA-MS/MS (acylcarnitines, lipids, and hexose) and LC/MS (amino acids, biogenic amines) using an AB SCIEX 4000 QTrap^®^ mass spectrometer (AB SCIEX, Darmstadt, Germany) with electrospray ionization. The fully automated assays were performed on a double-filter 96-well plate kit containing stable isotope-labeled internal standards for the quantification of amino acids, biogenic amines, acylcarnitines, lipids and hexose. Mass chromatograms were analyzed using BIOCRATES software (BIOCRATES Life Science AG). Detailed information regarding experimental metabolomics measurement technique, including internal standards, calculation of metabolite concentrations, analytical variance as well as accuracy of measurements is given elsewhere [25].

### Statistical Analysis

To compare differences among lipolysis groups, data were subjected to ANOVA using the MIXED procedure of SAS (version 9.3; SAS Inst. Inc., Cary, NC, USA). Means were reported as least-squares means ± standard error of the mean (SEM) and P≤0.05 and P≤0.10 were defined as significance and trend, respectively. For each response variable tested, the model included the fixed effects of mobilization and parity. As no differences in milk parameters, NEFA and RQUICKI were observed among breeds, the breed was considered as a random effect in the model. Degrees of freedom were approximated using Kenward-Rogers method (ddfm = kr). Associations among NEFA, RQUICKI and the determined serum metabolites were studied by performing a Pearson correlation analysis (PROC CORR of SAS).

## Results

### Differences in Lipolysis and Estimated Insulin Sensitivity among Groups

We used differences in NEFA concentration to distinguish among low, medium and high lipolysis cows (*P* < 0.001) with average NEFA concentrations of 0.23, 0.51, and 0.96 mmol/L for low, medium, and high group, respectively (S2 Table). While glucose concentrations did not differ, an impaired estimated insulin sensitivity, as indicated by lower (*P* < 0.001) RQUICKI values, was determined for the high group (0.39) compared to low (0.52), with the medium cows (0.46) showing intermediate values.

### Differences in Metabolome Profiling among Groups

The MS/MS targeted analysis provided results for 142 metabolites (Tables 1–3). Besides 12 lysophosphatidylcholines, 72 phosphatidylcholines, 15 sphingomyelins, 10 acylcarnitines and the sum of hexoses, 21 amino acids and 11 biogenic amines were detected using the Absolute-IDQ p180 kit.

**Table 1:** Concentrations of lysophosphatidylcholines, phosphatidylcholines and sphingomyelins in the serum of cows differing in the degree of lipolysis and parity.

Metabolite (μmol/l)	Lipolysis			SEM	*P*-Value^1^	
	LOW	MEDIUM	HIGH		Parity	Lipolysis
**Lysophosphatidyl-cholines**						
lysoPC a C16:0	24.4^b^	26.1^a^^b^	30.6^a^	0.945	0.66	0.016
lysoPC a C16:1	1.5	1.6	1.5	0.058	0.67	0.87
lysoPC a C17:0	2.2^a^	1.6^b^	1.7^b^	0.073	0.019	0.001
lysoPC a C18:0	20.8	18.4	21.0	0.587	0.58	0.19
lysoPC a C18:1	12.8	13.8	15.1	0.450	0.95	0.12
lysoPC a C18:2	22.2	24.4	24.9	0.850	0.08	0.35
lysoPC a C20:3	1.2	1.2	1.0	0.073	0.48	0.45
lysoPC a C20:4	1.6	1.8	1.6	0.073	0.14	0.61
lysoPC a C26:0	0.129	0.114	0.118	0.004	0.79	0.32
lysoPC a C26:1	0.071	0.071	0.081	0.003	0.19	0.21
lysoPC a C28:0	0.304	0.306	0.329	0.010	0.87	0.55
lysoPC a C28:1	0.575	0.568	0.627	0.06	0.65	0.64
**Phosphatidylcholines**						
PC aa C28:1	2.3^b^	2.3^a^^b^	2.8^a^	0.119	0.14	0.094
PC aa C30:0	3.1^b^	3.4^b^	4.2^a^	0.141	0.24	0.002
PC aa C30:2	0.80	0.87	1.02	0.048	0.49	0.18
PC aa C32:0	6.0^b^	6.1^a^^b^	7.2^a^	0.235	0.14	0.038
PC aa C32:1	6.4^b^	7.5^a^^b^	8.5^a^	0.316	0.83	0.020
PC aa C32:2	7.3	7.5	8.9	0.417	0.29	0.25
PC aa C32:3	15.5	15.1	17.6	0.955	0.22	0.57
PC aa C34:1	75.9^b^	82.6^b^	107.4^a^	3.68	0.98	<0.001
PC aa C34:2	147.5^b^	147.6^b^	188.5^a^	4.95	0.32	<0.001
PC aa C34:3	27.4^b^	29.7^b^	36.8^a^	1.21	0.75	0.001
PC aa C34:4	4.1	4.2	4.5	0.231	0.45	0.75
PC aa C36:0	8.0	8.0	8.4	0.336	0.87	0.88
PC aa C36:1	95.3^a^^b^	86.2^b^	108.2^a^	3.46	0.96	0.047
PC aa C36:2	181.5^a^^b^	162.7^b^	195.8^a^	5.14	0.53	0.057
PC aa C36:3	82.7^a^^b^	77.8^b^	94.0^a^	2.89	0.82	0.079
PC aa C36:4	29.7^b^	31.7^a^^b^	37.9^a^	1.38	0.41	0.026
PC aa C36:5	9.4^b^	12.0^a^^b^	13.1^a^	0.67	0.68	0.056
PC aa C36:6	2.7	3.1	3.4	0.19	0.89	0.28
PC aa C38:0	3.0	2.6	2.6	0.14	0.91	0.44
PC aa C38:1	4.4	3.6	4.2	0.23	0.93	0.48
PC aa C38:3	28.1	24.0	26.0	1.78	0.39	0.70
PC aa C38:4	32.6	32.6	35.5	1.66	0.40	0.73
PC aa C38:5	26.8	31.1	34.9	1.80	0.27	0.15
PC aa C38:6	5.5	6.5	7.1	0.38	0.09	0.16
PC aa C40:2	0.44	0.40	0.40	0.021	0.085	0.61
PC aa C40:3	2.8^a^	2.1^a^^b^	1.7^b^	0.191	0.91	0.036
PC aa C40:4	9.1	7.2	7.2	0.447	0.22	0.15
PC aa C40:5	23.1	22.6	27.5	0.460	0.34	0.22
PC aa C40:6	6.8	6.7	7.9	0.006	0.055	0.47
PC aa C42:0	0.090	0.113	0.099	0.008	0.0076	0.22
PC aa C42:1	0.12	0.14	0.13	0.011	0.017	0.40
PC aa C42:2	0.195	0.188	0.196	0.067	0.009	0.90
PC aa C42:4	0.20	0.18	0.18	0.029	<0.001	0.42
PC aa C42:5	1.21^a^	0.94^a^^b^	0.90^b^	0.067	0.087	0.089
PC aa C42:6	0.61^a^	0.49^a^^b^	0.46^b^	0.029	0.043	0.046
PC ae C30:0	0.65	0.68	0.77	0.026	0.58	0.14
PC ae C30:1	1.9	1.9	2.3	0.092	0.66	0.14
PC ae C30:2	0.41	0.42	0.46	0.019	0.88	0.54
PC ae C32:1	2.7	2.8	3.1	0.101	0.55	0.16
PC ae C32:2	4.2	4.2	5.2	0.230	0.66	0.18
PC ae C34:0	2.7	2.3	2.4	0.104	0.45	0.32
PC ae C34:1	12.2	11.8	13.3	0.406	0.38	0.34
PC ae C34:2	18.4	18.0	21.0	0.687	0.52	0.15
PC ae C34:3	19.9	20.6	22.7	0.978	0.51	0.46
PC ae C36:0	1.7	1.6	1.6	0.072	0.77	0.93
PC ae C36:1	16.8	14.6	16.9	0.570	0.20	0.24
PC ae C36:2	27.6^a^	21.1^b^	24.9^a^^b^	1.01	0.22	0.034
PC ae C36:3	11.3	10.1	11.4	0.430	0.23	0.49
PC ae C36:4	8.1	8.6	9.1	0.486	0.69	0.67
PC ae C36:5	5.0	5.7	6.3	0.286	0.38	0.17
PC ae C38:0	1.8	1.9	2.0	0.077	0.74	0.46
PC ae C38:1	4.4	3.5	4.1	0.195	0.100	0.27
PC ae C38:2	4.6	3.8	4.4	0.196	0.15	0.38
PC ae C38:3	4.8	4.0	4.3	0.200	0.18	0.26
PC ae C38:4	4.2	3.8	4.0	0.172	0.009	0.74
PC ae C38:5	3.7	3.7	3.9	0.179	0.050	0.84
PC ae C38:6	3.6	4.1	4.5	0.229	0.63	0.27
PC ae C40:1	0.82	0.74	0.75	0.038	0.022	0.59
PC ae C40:2	1.5	1.5	1.5	0.065	0.15	0.91
PC ae C40:3	1.7	1.7	1.8	0.104	0.013	0.90
PC ae C40:4	1.9	2.1	2.2	0.136	0.002	0.59
PC ae C40:5	2.8	2.8	2.9	0.144	0.012	0.88
PC ae C40:6	1.5	1.4	1.5	0.067	0.026	0.86
PC ae C42:1	0.28	0.28	0.29	0.013	0.013	0.95
PC ae C42:2	0.35	0.36	0.36	0.018	0.008	0.99
PC ae C42:3	0.52	0.48	0.48	0.027	<0.001	0.67
PC ae C42:4	1.07	1.10	1.28	0.086	<0.001	0.37
PC ae C42:5	1.05	1.00	1.11	0.051	0.004	0.64
PC ae C44:3	0.103	0.110	0.114	0.006	0.003	0.70
PC ae C44:4	0.13	0.13	0.12	0.006	<0.001	0.65
PC ae C44:5	0.092	0.089	0.087	0.004	0.001	0.78
PC ae C44:6	0.077	0.077	0.070	0.002	0.009	0.33
**Sphingomyelins**						
SM(OH) C14:1	12.6	12.2	14.5	0.532	0.33	0.23
SM(OH) C16:1	8.1	7.1	8.3	0.317	0.32	0.36
SM(OH) C22:1	18.8^b^	21.8^a^	26.9^a^	0.936	0.40	<0.001
SM(OH) C22:2	8.1^b^	8.5^b^	10.2^a^	0.308	0.55	0.008
SM(OH) C24:1	1.8	1.8	2.0	0.062	0.80	0.31
SM C16:0	99.8^b^	99.0^b^	122.7^a^	3.76	0.81	0.009
SM C16:1	10.1	10.0	11.8	0.444	0.93	0.21
SM C18:0	13.0^b^	12.2^b^	15.6^a^	0.439	0.95	0.003
SM C18:1	4.2^b^	3.9^b^	4.9^a^	0.152	0.43	0.024
SM C20:2	0.14^b^	0.12^b^	0.26^a^	0.018	0.86	0.004
SM C22:3	0.12^a^^b^	0.04^b^	0.15^a^	0.019	0.74	0.08
SM C24:0	35.2	31.2	33.6	1.36	0.72	0.58
SM C24:1	10.6^b^	11.6^b^	14.1^a^	0.466	0.30	0.001
SM C26:0	0.48	0.48	0.49	0.020	0.99	0.97
SM C26:1	0.29	0.29	0.29	0.016	0.096	0.99

^1^ Effect of parity included primiparous (n = 12) vs. multiparous (n = 18) cows, and effect of lipolysis included low (n = 10) vs. medium (n = 8) vs. high (n = 12) lipolysis groups of cows.

^ab^ indicate differences among LS means at *P* ≤ 0.05.

**Table 2:** Concentrations of acylcarnitines in the serum of cows differing in the degree of lipolysis and parity.

Metabolite (μmol/l)	Lipolysis			SEM	P-Value^1^	
	LOW	MEDIUM	HIGH		Parity	Lipolysis
C0	5.5^a^	4.8^a^^b^	4.5^b^	0.295	<0.001	0.068
C2	1.9^b^	2.9^a^	3.1^a^	0.217	0.002	0.011
C3	0.27^a^	0.24^a^^b^	0.20^b^	0.015	<0.001	0.048
C3-DC	0.06	0.07	0.08	0.005	0.42	0.16
C4	0.10	0.09	0.10	0.006	<0.001	0.83
C5	0.07	0.08	0.07	0.005	0.002	0.60
C14:1	0.06^b^	0.06^b^	0.07^a^	0.002	0.18	0.002
C16	0.01^c^	0.02^b^	0.03^a^	0.002	0.76	<0.001
C18	0.02^c^	0.03^b^	0.05^a^	0.003	0.95	<0.001
C18:1	0.03^b^	0.03^b^	0.05^a^	0.295	0.66	<0.001

^1^ Effect of parity included primiparous (n = 12) vs. multiparous (n = 18) cows, and effect of lipolysis included low (n = 10) vs. medium (n = 8) vs. high (n = 12) lipolysis groups of cows.

^ab^ indicate differences among LS means at *P* ≤ 0.05.

**Table 3:** Serum amino acids, biogenic amines, and sum of hexoses in cows with low (n = 10), medium (n = 8), and high (n = 12) fat mobilization after calving.

Metabolite (μmol/l)	Lipolysis			SEM	*P*-Value^1^	
	LOW	MEDIUM	HIGH		Parity	Lipolysis
**Amino Acids**						
Alanine	275.3	269.9	259.9	10.00	0.055	0.80
Arginine	165.4	172.6	161.6	4.85	0.26	0.73
Asparagine	37.0	38.2	32.2	1.23	0.058	0.12
Aspartate	10.0^a^^b^	11.8^a^	8.1^b^	0.652	0.051	0.091
Citrulline	63.0	56.4	59.3	3.01	0.38	0.72
Glutamine	230.5	239.9	231.1	8.33	0.052	0.92
Glutamate	76.1	73.6	77.5	2.46	0.25	0.84
Glycine	441.7^b^	617.1^a^	613.4^a^	25.53	0.79	0.004
Histidine	43.8	42.4	42.3	1.93	0.29	0.94
Isoleucine	156.7	186.1	201.5	10.49	0.92	0.20
Leucine	174.8	201.0	192.3	8.36	0.99	0.51
Lysine	135.2	147.5	128.2	5.21	0.14	0.45
Methionine	19.6	21.1	18.1	0.856	0.25	0.44
Ornithine	44.8	45.8	38.7	1.74	0.52	0.15
Phenylalanine	61.2	67.3	64.7	1.50	0.26	0.30
Proline	125.0	138.9	119.9	3.93	0.095	0.24
Serine	144.4	140.7	115.7	6.57	0.034	0.13
Threonine	93.2^a^	96.2^a^	71.6^b^	4.14	0.36	0.034
Tryptophane	51.9	53.0	44.9	1.84	0.50	0.13
Tyrosine	65.4	62.0	56.8	2.10	0.12	0.23
Valine	272.2	303.1	304.8	10.05	0.82	0.35
**Biogenic Amines**						
Asymmetric dimethylarginine	0.59	0.50	0.47	0.042	0.73	0.51
α-Aminoadipic acid	3.2	3.6	3.7	0.220	0.41	0.63
Carnosine	6.9	7.6	8.1	0.361	0.20	0.34
Creatinine	98.0	94.6	103.3	3.46	0.55	0.53
Kynurenine	10.9	9.5	9.3	0.428	0.48	0.24
Methionine-Sulfoxide	8.1	9.1	7.9	0.355	<0.001	0.30
Sarcosine	1.0	1.1	1.0	0.063	0.69	0.53
Serotonine	2.4	4.5	3.9	0.471	0.17	0.23
trans-4-Hydroxyproline	17.3	18.6	19.1	0.808	0.004	0.53
Taurine	33.2^b^	54.4^a^	46.7^a^	2.69	0.47	0.005
Symmetric dimethylarginine	0.62	0.55	0.67	0.026	0.36	0.37
**Sum of hexoses**	3528.4	3245.2	3285.9	77.10	0.35	0.26

^1^ Effect of parity included primiparous (n = 12) vs. multiparous (n = 18) cows, and effect of lipolysis included low (n = 10) vs. medium (n = 8) vs. high (n = 12) lipolysis groups of cows.

^ab^ indicate differences among LS means of various lipolysis groups at *P* ≤ 0.05.

Of the detected metabolites, the greatest effect of lipid mobilization was detected for serum profiles of acylcarnitines, sphingomyelins, and phosphatidylcholines with diacyl-residues. High lipomobilization was associated with an increase in serum long-chain lysophosphatidylcholine C16:0 compared to the low group (*P* = 0.02), whereas lysophosphatidylchloine C17:0 was decreased in cows of medium and high mobilization compared to low lipomobilization (*P* < 0.01). Additionally, lysophosphatidylcholine C17:0 differed between parities, showing lower concentrations in multiparous compared to primiparous cows (*P* = 0.02).

Serum concentration of several phosphatidylcholines with diacyl residues up to C36 (C28:1, C30:0, C32:0, C32:1, C34:1, C34:2, C34:3, C36:1, C36:2, C36:3, C36:4, C36:5) were increased (*P* ≤ 0.09) in high mobilizing cows, whereas the opposite effect was noticed for phosphatidylcholines with longer chains (C40:3, C42:5, C42:6; P≤0.09). Interestingly, only one of the 37 detected phosphatidylcholines with diacyl residues showed different concentrations among mobilization groups, with higher concentrations of PC ae C36:2 in cows of the low group compared to cows of the medium group (*P* = 0.03).

A significant parity effect was found for PC aa C42:0, PC aa C42:1, PC aa C42:2, PC aa C42:4, PC aa C42:6, PC ae C38:4, PC ae C40:1, PC ae C40:6 PC ae C40:4, PC ae C40:5, PC ae C40:6, PC ae C42:1, PC ae C42:2, PC ae C42:3, PC ae C42:4, PC ae C42:5, PC ae C44:3, PC ae C44:4, PC ae C44:5, and PC ae C44:6, showing lower concentrations in multiparous compared to primiparous cows (*P* < 0.05).

There was an effect of mobilization on serum concentrations of certain long-chain sphingomyelins (SM(OH) C22:1, SM(OH) C22:2, SM C16:0, SM C18:0, SM C18:1, SM C20:2, SM C22:3 and SM C24:1), indicating that high lipid mobilization enhanced the concentrations of these sphingomyelins (*P* ≤ 0.08).

Of the 10 detected acylcarnitines, 7 showed differing concentrations between the mobilization groups (Table 2). While concentrations of free carnitine (C0) and propionylcarnitine (C3) were reduced with increasing degree of fat mobilization (P≤0.07), the opposite effect was noticed for acetylcarnitine (C2), myristoleylcarnitine (C14:1), palmitoylcarnitine (C16), stearylcarnitine (C18) and oleylcarnitine (C18:1) (*P* ≤ 0.01). Furthermore, concentrations of several acylcarnitines (C0, C2, C3, C4 and C5) were decreased in multiparous compared to primiparous cows (*P* < 0.01).

Similar to glucose, the sum of hexoses did not differ among mobilization groups. Of the targeted amino acids, high lipid mobilizing cows showed greater concentrations of glycine (*P* < 0.01), whereas the opposite effect was noticed for threonine in the respective cow group compared to medium and low lipid mobilizing cows (*P* = 0.03, Table 3). Aspartate tended to be increased in cows of the medium group compared to the high lipid mobilizing cow group (*P* = 0.09). Of the detected biogenic amines differences between mobilization groups were only detected for taurine, showing the lowest concentrations in the low lipid mobilizing cow group compared to the two other groups (*P* < 0.01). Primiparous cows showed higher concentrations of serine, methionine-sulfoxide and trans-4-hydroxyproline compared to multiparous cows (*P* < 0.05).

### Correlations between RQUICKI, NEFA and Serum Metabolites

**Fig 1:**
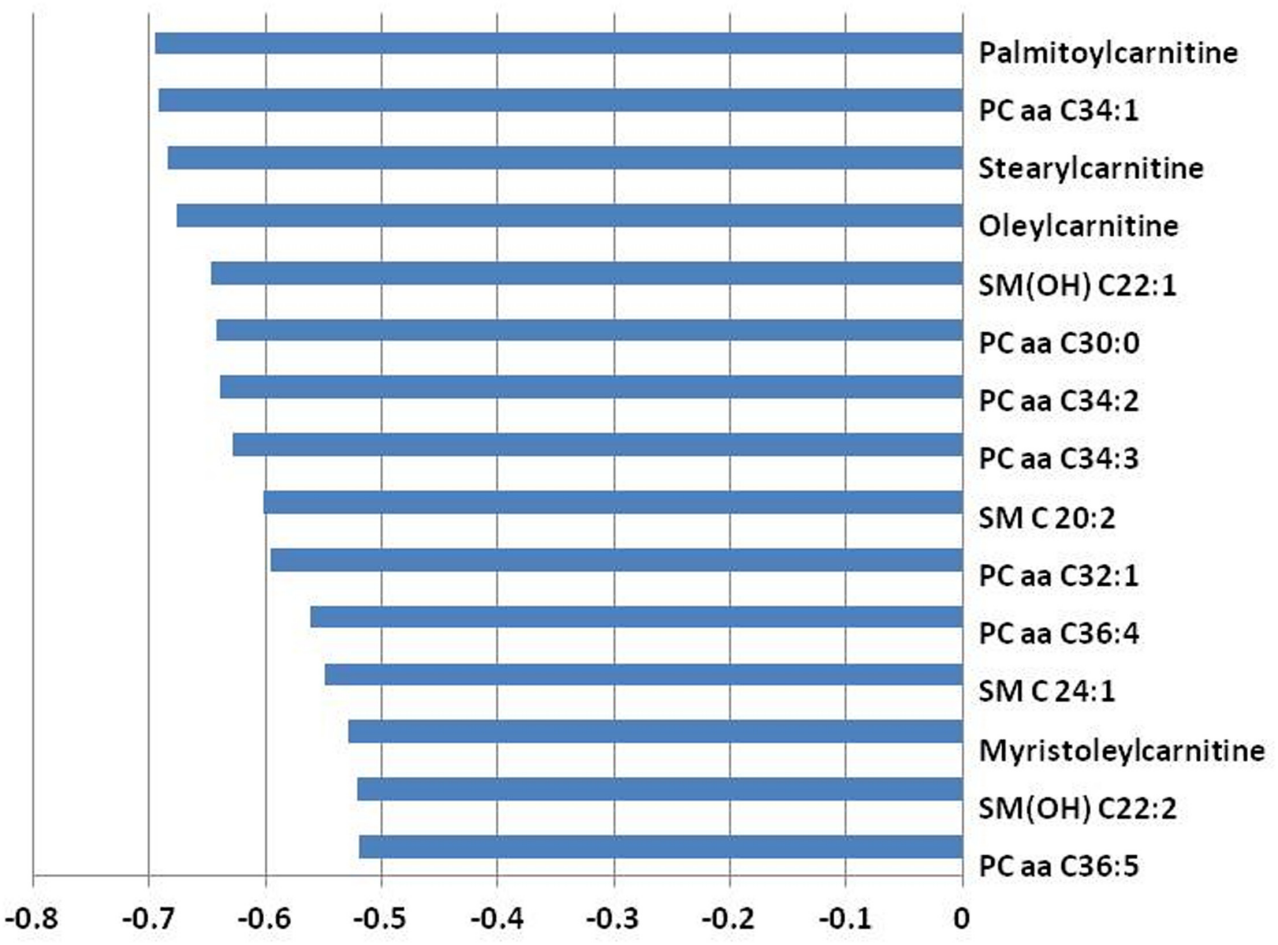
A bar graph showing the top 15 metabolites correlating with RQUICKI. The X-axis indicates Pearson correlation coefficients.

**Fig 2:**
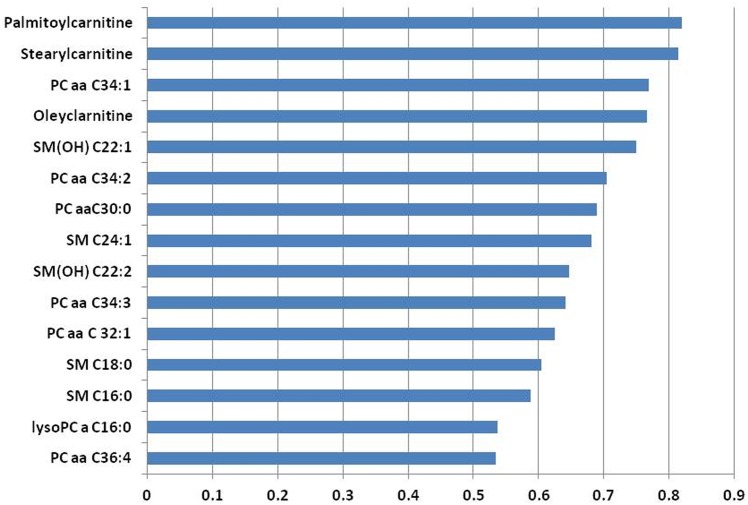
A bar graph showing the top 15 metabolites correlating with nonesterified fatty acids. The X-axis indicates Pearson correlation coefficients.

Fig 1 presents the 15 serum metabolites showing the highest correlation coefficients (r <-0.5, *P* < 0.01) with RQUCIKI. Highest correlations among RQUICKI and serum metabolites have been determined for acylcarnitines (i.e., palmitoylcarnitine, stearylcarnitine, oleylcarnitine, and myristoleylcarnitine), phosphatidylcholines (i.e., PC aa C34:1, PC aa C30:0, PC aa C34:2, PC aa C34:3, PC aa C32:1, PC aa C36:4, and PC aa C36:5), and sphingomyelins (i.e., SM(OH) C22:1, SM C20:2, SM C24:1, and SM(OH) C22:2). In accordance, highest correlations among NEFA (r > 0.5, *P* < 0.01) and serum metabolites were found for acylcarnitines (i.e., palmitylcarnitine, stearylcarnitine and oleylcarnitine), phosphatidylcholines (i.e., PC aa C34:1, PC aa C34:2, PC aa C30:0, PC aa C34:3, PC aa C32:1, and PC aa C36:4), sphingomyelins (i.e., SM(OH) C22:1, SM C24:1, SM(OH) C22:2, SM C18:0, SM C16:0) and for the lysophosphatidylcholine a C16:0 (Fig 2).

## Discussion

To gain a deeper understanding about the alterations of blood metabolites in relation to lipomobilization in the early postpartum period and to identify serum biomarkers that distinguish cows with high lipid mobilization from those with medium and low lipid mobilization, this study evaluated the serum metabolome profiles of dairy cows with varying degrees of lipolysis on d 21 after parturition. The metabolomic profiling was part of a larger study [10] conducted in dairy cows kept under the same feeding and management conditions. Despite similar milk production those cows showed differences in several endocrine and metabolic responses postpartum, especially in terms of ciruclating NEFA and estimated insulin function. Thus, we extended our previous study by using a targeted metabolomics approach, to gain a more comprehensive view on the observed variability among individual cows, and particularly to enable a better understanding of the underlying mechanisms of excessive lipolysis.

Most importantly, cows of different lipolysis groups showed clearly discernible changes in serum acylcarnitine, sphingomyeline, and phospholipid metabolome profiles. Choline-containing phospholipids (mainly phosphatidylcholines) play a pivotal role in lipid absorption and transport, cell-signaling and synthesis of lipoproteins [26]. For instance, bovine VLDL phospholipids are mainly phosphatidylcholines, with smaller proportions of sphingomyelins and phosphatidylethanolamine. Although the concentration of VLDL in bovine plasma is very low, they are essential primary sources of lipids for extrahephatic tissues, especially in the early lactation period [27, 28]. The accelareted mobilization of body fat stores during early lactation enhances the release of NEFA, which are bound to albumine to enable transportation to various tissues. Nonesterified fatty acids are taken up by the liver in proportion to their circulating concentration and are either oxidized to CO_2_ or ketone bodies or esterified to TAG [29]. The liver normally packages the TAG in VLDL, which are secreted into the bloodstream and transported to the mammary tissue; however, a sudden increase in plasma NEFA may not be adequately processed by the bovine liver [30, 31]. Phosphatidylcholines are essential for the synthesis of VLDL, thus being responsible for the export of TAG [14]. In association with cholesterol, they surround the hydrophobic core of TAG by forming a monolayer on the lipoprotein surface [28]. Synthesis of phosphatidylcholines occurs primarily in the hepatocytes, by either sequential methylation of phosphatidylethanolamine or via the CDP-choline pathway, with the latter representing the major pathway [14]. As the rate of choline-containing phospholipid synthesis is decreased when choline availability is restricted—due to its requirement in the CDP-pathway—low levels of choline are often blamed for the accumulation of TAG in the liver and development of fatty liver disease in dairy cattle [32]. Thus, a massive mobilization of fatty acids that is accompanied by lipotropic factor deficiency (i.e. choline and methionine), declines the rate of packaging of TAG into VLDL and results in a reduced export from the liver. In further consequence, TAG accumulate in the liver and may promote fatty liver development [31, 32]. To sum up, incapability to rapidly enhance VLDL production and export from the liver may be due to deficient synthesis of the essential components phospholipids (particularly phosphatidylcholines), cholesterol, or apolipoproteins.

An interesting and novel aspect of the present study was that from the detected phosphatidylcholines mainly those with diacyl-residues showed differences among mobilization groups. This might be attributed to their different functions, as diacyl-phosphatidylcholines are essential for hepatic VLDL-secretion, whereas acyl-alkyl-phosphatidylcholines seem to primarily act as serum antioxidants [16]. From the 35 detected PC with aa, the following 15 PC differed among mobilization groups of cows: PC aa C28:1, PC aa C30:0, PC aa C32:0, PC aa C32:1, PC aa C34:1, PC aa C34:2, PC aa C34:3, PC aa C36:1, PC aa C36:2, PC aa C36:3, PC aa C36:4, PC aa C36:5, PC aa C40:3, PC aa C42:5, and PC aa C42:6. Although changes of phosphatidylcholine and sphingomyelin blood levels have been previously reported to be linked to metabolic disorders [16], the potential association of the listed phosphatidylcholines with lipid mobilization seems intriguing. Because they are required to assemble and secrete VLDL [17], a reduced PC concentration may trigger the accumulation of TAG in the liver by impairing their export from hepatocytes [18]. The observed changes in PC levels demonstrate an increased quantity of PC containing fatty acid components ranging from 28 to 36 carbons, while PC carrying larger fatty acid moieties (40 carbons) were reduced. A similar remodeled PC distribution was recently found by Imhasly et al. [33]. In their study, PC with 30 and 32 carbons were increased, whereas those containing larger chains (≥ 36 carbons) were reduced in cows diagnosed with hepatic lipidosis. In agreement to a recent study conducted in humans [14] these results lead to the suggestion, that lipids with a shorter chain length may aggravate high lipid mobilization, whereas those containing larger fatty acid moieties may offer protection. However, the detailed mechanisms remain unclear so far and require further investigations.

Besides phosphatidylcholines, also some lysophosphatidylcholines were affected by mobilization. These phospholipid groups are important structural components of plasma lipoproteins and cell membranes, being involved in the regulation of cell function, membrane protein trafficking and inflammation [34]. Lysophospholipids are a component of oxidized LDL, that are derived from partial hydrolysis of phosphatidylcholines [35]. Saturated lysophospholipids were recognized to exert pro-inflammatory effects and impair insulin signalling [36, 37]. In addition, alterations in blood phospholipid and lysophospholipid profiles are linked to insulin resistance [14, 38]. Overall, the observed findings require further investigations to understand if it has a causal relationship with the lipomobilization or rather constitutes a consequence thereof.

Sphingolipids are a class of lipids, showing several functions, such as stabilization of the membrane structure, and cell-to-cell recognition and signalling [39, 40]. They are composed of a backbone of sphinganine, which is modified to generate ceramide and more complex compounds, such as sphingomyelin [41]. The synthesis of sphingolipids in the endoplasmatic reticulum generates sphingolipids of various acyl chain length from non-sphingolipid precursors [34]. Among the vast diversity of sphingolipids alterations in the metabolism of specific sphingolipids, i.e. C16:0 species, have been reported to play a major role in the progression of insulin resistance in humans [41]. For instance, Hanamatsu et al. [17] observed elevated levels of certain sphingomyelins in obese humans and found close correlations with the parameters of obesity, insulin resistance, liver function, and lipid metabolism. It is believed that especially C16:0-ceramide mediates the key pathophysiology of disturbed insulin function [41, 42], which seems to be attributable to its proapoptotic effect on membranes [41]. We also observed high correlations between RQUICKI and the concentration of certain sphingomyelins in the present study. Thereby, it is noteworthy that more than the half of the detected sphingomyelins were increased ranging from 17 to 86% in cows experiencing the highest degree of lipomobilization. Recent research conducted by Rico et al. [6] corroborate the present findings. These authors observed elevated plasma concentrations of sphingolipids—which derive from the hydrolysis of sphingomyelins—in cows with greater adiposity and higher plasma NEFA. This suggests that these sphingomyelins might mediate the progression of insulin resistance in dairy cows early postpartum. In this regard, increasing evidence supports a contributive function of long-chain saturated fatty acids to a reduced whole-body insulin sensitivity [43, 44], whereby especially sphingolipids seem to affect the ability of surplus saturated fatty acyl-CoA to inhibit sensitivity to insulin [45]. Overall, the present study reveals alterations in the serum phospholipidome in dairy cows and demonstrates an association in the increase in serum sphingolipids with a disturbed insulin function during the early postpartal period. Thus, the current findings suggest a constant alteration in the synthesis or breakdown of sphingolipids and phospholipids in cows experiencing excessive degrees of lipolysis.

Carnitine is required for the transport of long-chain fatty acids (LCFA) into the mitochondria supporting ß-oxidation [46]. Thus, carnitine is not only an important component of fatty acid oxidation, but also of hepatic ketogenesis [47]. The functions of carnitine are accomplished through the action of acyltransferases, which produce carnityl esters (acylcarnitines) [46]. In the present study, high mobilizing cows had elevated concentrations of acetylcarnitine (C2), myristoleylcarnitine (C14:1), stearoylcarnitine (C18) and oleoylcarnitine (C18:1), whereas concentrations of free carnitine (C0) and propionylcarnitine (C3) were reduced in these cows compared to low mobilizing cows. Accordingly, similar changes in plasma concentrations of carnitine and acylcarnitines were observed in diabetics compared to healthy humans [18]. Those authors detected enhanced acetylcarnitine, reduced propionylcarnitine as well as elevated concentrations of stearoylcarnitine and oleoylcarnitine in diabetics. Despite these evidences, molecular links between those changes in acetylcarnitines and insulin signaling pathways remain unresolved so far. However, according to Adams et al. [18], mainly the stimulating effect of acylcarnitines on proinflammatory pathways which promote insulin resistance, seem to play a pivotal role. Furthermore, several studies in humans have reported reduced levels of free carnitine concurrent with increased levels of acylcarnitine during diabetic and fasting ketosis [48, 49]. Overall, the reduced concentrations of free carnitine in high-mobilizing cows seem to derive from the enhanced need of carnitine for the transport of fatty acids into the mitochondrial matrix due to the high mobilization from the stored lipids to generate metabolic energy. Having in mind the pivotal role of carnitine in hepatic fatty acid oxidation, our results suggest that the carnitine status might influence the degree of liver lipid accumulation in peripaturient dairy cows. For example, Grum et al. [50] observed a decreased liver TAG accumulation during the transition period due to increased liver carnitine concentrations prepartum.

Perturbations of the tricarbon cylce (TCA) itself might be one explanation for the incomplete oxidation of the LCFA via ß-oxidation in tissues of high-mobilizing cows, as indicated by elevated levels of acylcarnitines that carry fatty acid moieties with ≥14 carbons. Overall, elevated levels of acetyl-CoA produced from oxidation of NEFA exceeding the capacity of the TCA, should increase their conversion to acetylcarnitine. Likewise, several authors [18, 51, 52] have observed increased acetylcarnitine concentrations in humans with impaired glucose tolerance and type 2 diabetes, respectively. Furthermore, Adams et al. [18] reported reduced plasma propionylcarnitine, indicating that increasing plasma markers of incomplete LCFA ß-oxidation are related to enhanced levels of acetylcarnitine, accompanied by decreased propionylcarnitine concentrations. Consequently, it might be assumed that a high lipid-mobilization-associated decrease in tissue pools of anaplerotic propionyl-CoA compromises the generation of TCA cycle intermediates and thus affects oxidative disposal of acetyl-CoA and LCFA-CoA in mitochondria [18]. Nevertheless, whether fatty acylcarnitines could be used as biomarkers for pre-onset insulin resistance and hyperlipidemia in transition dairy cows is unknown.

Here, we report high correlations between saturated long-chain acylcarnitines (e.g. palmitoylcarnitine and stearoylcarnitine) and the estimated insulin resistance as indicated by RQUICKI (r ~—0.7) and NEFA (r ~ 0.8). Thus, these sphingolipids and acyl-carnitines or their overall profiles may serve as novel biomarkers for insulin resistance and thus lipid mobilization in dairy cows.

Three of the 37 differing metabolites were the amino acids glycine, threonine and aspartate. Foremost, glycine, as the most abundant amino acid in blood, was enhanced in high lipid mobilizing cows compared to the low mobilizing cows. Glycine has been reported to be involved in serine and threonine metabolism [12], and appears to be associated with metabolic and chronic inflammatory conditions [53, 54]. The latter were reported to occur during severe lipid mobilization in cows [33]. Furthermore, glycine and other amino acids might directly cause insulin resistance by disrupting insulin signaling [55]. Indeed, glycin was also found to be significantly altered in impaired glucose tolerance in humans [51]. Thus, it might also serve as an early marker of insuline resistance [56]. An additional explanation might be the secretion of glycine by the skeletal muscles to maintain blood glucose levels due to the predominance of catabolic metabolism [57]. The catabolism of protein is known to contribute to the maintenance of normal blood glucose levels in cows. Thus, glucogenic amino acids, such as glycine, valine, threonine and aspartate are metabolized into phosphoenolpyruvate to provide glucose through gluconeogenesis [58]. Indeed, besides glycine, the analysis also revealed differences in the concentrations of other glucogenic amino acids, such as asparate and threonine among different lipolysis groups. However, further studies are required to elucidate the mechanisms for the differences in these amino acids in relation to lipid mobilization in postpartal diary cows.

Previous reports regarding the effect of parity on metabolic and endocrine changes early postpartum are scarce and equivocal [59, 60]. Overall, the higher NEFA, lower glucose and RQUCIKI found in multiparous cows suggest an increased risk for high lipid mobilization in the respective cows [10]. This is further supported by several changes observed in the serum metabolome, like a reduction in lyso PC a C17:0, PC aa C42:5, PC aa C42:6, free carnitine and propionylcarnitine. As these reductions have also been found for high vs. low fat-mobilizing cows, the higher mobilization of multiparous cows as compared with primiparous cows seems to be instrumental for these changes rather than parity per se. Although multiparous cows have shown a higher feed intake compared to primiparous cows, their higher milk production potential seems to predisposes them to suffer from a stronger negative energy balance than primiparous cows. Indeed, also prevalence of ketosis and other diseases associated with excessive lipolysis, have been reported to be higher in multiparous compared to primiparous cows [61–63].

## Conclusions

In summary, the study revealed 37 metabolites associated with excessive lipolysis which may be used as biomarkers to identify cows with high lipid mobilization. Specifically, PC carrying fatty acid moieties ranging from 28 to 36 carbons were enhanced in high lipid mobilizing cows, whereas the quantity of PC containing larger fatty acid components (≥40 carbons) was reduced. This suggests that elevated concentrations of lipids with a shorter chain length may reflect excessive lipid mobilization, whereas elevated long-chain fatty acids may stand for low lipid mobilization. Further changes in the metabolite profile such as an increase in sphingolipids and a reduction in free carnitine and propionylcarnitine may also belong to metabolic pathways that are modified during excessive lipid mobilization in early-lactating cows. These novel candidates could be useful in practice to identify high-risk cows at an earlier stage and use them to alleviate or prevent the onset of diseases associated with excessive lipid mobilization in cows.

## Supporting Information

S1 Table Ingredients and chemical composition of the fresh-lactating diet. (DOCX)

S2 Table Serum NEFA and insulin sensitivity variables in cows differing in the degree of lipolysis on d 21 postpartum [adapted from 10]. (DOCX)
