# Supplementary material for: Alterations of the Lipid Metabolome in Dairy Cows Experiencing Excessive Lipolysis Early Postpartum
Source: PLoS One. 2016 Jul 6;11(7):e0158633. doi: 10.1371/journal.pone.0158633 (PMC4934687; doi:10.1371/journal.pone.0158633)
Supplement: S1 Table — (DOCX) [file pone.0158633.s001.docx]

**S1 Table. Ingredients and chemical composition of the fresh-lactating diet.**

| **Item** | **% of dry matter** |
| --- | --- |
| Ingredients |  |
| Meadow hay | 10.0 |
| Grass silage | 20.0 |
| Corn silage | 20.0 |
| Barley grain | 38.5 |
| Soybean meal | 5.23 |
| Rapeseed meal | 5.23 |
| Limestone | 0.58 |
| Salt | 0.23 |
| Vitamin-mineral premix^1^ | 0.23 |
| Nutrient composition |  |
| Dry matter, % | 65.4 |
| Ash | 7.9 |
| Crude protein (CP) | 15.3 |
| Neutral detergent fiber (NDF) | 32.4 |
| Acid detergent fiber (ADF) | 18.6 |
| Ether extract | 2.9 |
| Non-fiber carbohydrates (NFC)^2^ | 41.7 |
| Net energy lactation (NE_L_), MJ/kg | 6.56 |

^1^Contained Ca 6%, P 12%, Mg 10%, Na 8%, Mn 1,500 mg/kg, Zn 5,700 mg/kg, Cu 800 mg/kg, vitamin A 750,000 IU/kg, vitamin D3 75,000 IU/kg, vitamin E 3,000 mg/kg.

^2^Non-fiber carbohydrates= 100 - (% NDF + % CP + % ether extract + % ash).
