# Supplementary material for: Alterations of the Lipid Metabolome in Dairy Cows Experiencing Excessive Lipolysis Early Postpartum
Source: PLoS One. 2016 Jul 6;11(7):e0158633. doi: 10.1371/journal.pone.0158633 (PMC4934687; doi:10.1371/journal.pone.0158633)
Supplement: S2 Table — (DOCX) [file pone.0158633.s002.docx]

**S2 Table. Serum NEFA and insulin sensitivity variables in cows differing in the degree of lipolysis on d 21 postpartum [adapted from 10].**

| **Serum variable** | **Lipolysis** | | | | | **SEM** | | ***P*-Value^1^** | | |  |
| --- | --- | --- | --- | --- | --- | --- | --- | --- | --- | --- | --- |
|  | **LOW** | | **MEDIUM** | | **HIGH** |  | | **Parity** | | **Lipolysis** |  |
| NEFA, mmol/L | 0.23^c^ | 0.51^b^ | | 0.96^a^ | | | 0.041 | | <0.001 | <0.001 | |
| Glucose, mg/dL | 64.5 | 60.8 | | 59.2 | | | 1.28 | | 0.043 | 0.18 | |
| Insulin, µU/dL | 6.1^ab^ | 4.4^b^ | | 7.1^a^ | | | 0.43 | | 0.007 | 0.07 | |
| RQUICKI^2^ | 0.52^a^ | 0.46^b^ | | 0.39^c^ | | | 0.012 | | 0.010 | <0.001 | |

^1^ Effect of lipolysis (low (n = 10) vs. medium (n = 8) vs. high (n = 12) lipolysis groups) or parity (primiparous vs. multiparous).
^2^ RQUICKI = revised quantitative insulin sensitivity check index, calculated as 1/[log (glucose) + log (insulin) + log (NEFA)]), with lower values indicating lowered sensitivity to insulin.
^abc^ indicate differences among LS means at *P* ≤ 0.05.
